# Oxysterols and Retinal Microvascular Dysfunction as Early Risk Markers for Cardiovascular Disease in Normal, Ageing Individuals

**DOI:** 10.3390/antiox10111756

**Published:** 2021-11-03

**Authors:** Hala Shokr, Irundika HK Dias, Doina Gherghel

**Affiliations:** 1 Vascular Research Laboratory, College of Health and Life Sciences, Aston University, Birmingham B4 7ET, UK; Shokrh@aston.ac.uk; 2 Aston Medical Research Institute, Aston Medical School, Aston University, Birmingham B4 7ET, UK

**Keywords:** oxidative stress, oxysterols, cardiovascular disease risk

## Abstract

The aim of the present paper is to assess the relationship between oxysterol levels and retinal microvascular function in individuals of various age groups, free of clinically evident diseases. Forty-two apparently healthy individuals were included in the present study (group 1: 19–30 years, group 2: 31–50 years, and group 3: 51–70 years). Retinal microvascular function was assessed using the dynamic retinal vessel analyzer (DVA, IMEDOS GmbH, Jena, Germany). Fasting plasma was obtained from all subjects and quantification of monohydroxy and dihydroxy oxysterols assessment was performed using LC-MS/MS following reverse phase chromatography. A Griess assay was used to evaluate the Nitric Oxide (NO) concentration in all individuals. The glutathione redox ratio was also analyzed by means of whole blood glutathione recycling assay. In all participants, the levels of 7-Ketocholesterol, 25-hydroxycholesterol and 7β-hydroxycholesterol correlated significantly and positively with the time to maximum arteriolar dilation. In addition, 25-hydroxycholesterol and 7β-hydroxycholesterol negatively correlated to the percentage of maximum arteriolar dilation. A negative correlation was observed for 27-hydroxycholesterol and 7β-hydroxycholesterol with microvascular arteriolar constriction. These results suggest that, with age, abnormal oxysterol levels correlate with early changes in microvascular bed function. This relationship could signal early risk for cardiovascular diseases (CVDs) in an ageing population.

## 1. Introduction

The assessment of microvascular function gained a lot of clinical interest since endothelial dysfunction, the main culprit for CVDs, was found to occur earlier at the microvessel level than the macrovessel level [1]. Accordingly, many methods were developed to evaluate functional microvascular responses to biological stimuli in different vascular beds [2]. One of these methods, the dynamic retinal vessel analysis (DVA), was highlighted as one of the most sensitive and reliable methods to measure early vascular changes that point out early endothelial dysfunction and future risk of cardiovascular pathologies in individuals with and without overt clinical symptoms [3,4,5]. Moreover, it has also been shown that retinal microvascular function is modified by age [6]. Nevertheless, although this is a consistently demonstrable effect, little is known about what factors in particular directly determine this outcome. We have previously demonstrated that high levels of circulating reactive oxygen species (ROS) have a direct effect on retinal microvascular function [7,8]. Indeed, the evidence suggests that oxidative stress-induced cellular senescence is a key influencer of age-induced mechanical and structural vascular dysfunction [9,10,11,12] as well as of the development and progression of CVDs [13,14,15,16,17]. Additionally, high levels of ROS deplete the NO reserve, a well-known critical molecule in the regulation of vascular function and tone [18].

High levels of cholesterol are also an important factor affecting microvascular function at all levels, including the retinal microvascular function [8,19,20]. Cholesterol can be oxidized by non-enzymatic and enzymatic pathways [21,22]. The non-enzymatic pathway can be free-radical mediated and results in the formation of 7-hydroxycholesterol which is further oxidized to other oxysterols (7-ketocholesterol (7-KC), and 7β-hydroxycholesterol (7β-OHC) [23]. The enzymatic oxidation by cholesterol-hydroxylase enzymes produces 4β-hydroxycholesterol (4β-OHC), 7α-hydroxycholesterol, 24-hydroxycholesterol, 25-hydroxycholesterol (25-OHC), 27-hydroxycholesterol and dihydroxy oxysterols such as 7,25-hydroxycholesterol and 7,27-hydroxycholesterol (7,25-OHC) [24] and high levels of oxysterols in plasma have been highlighted as significant contributors to macrovessels atherogenesis and endothelial dysfunction [25,26,27]. Moreover, oxysterols, due to their marked pro-oxidant, proinflammatory and proapoptotic properties, have also been implicated in the pathogenesis of vascular ageing, [28]. Moreover, their effect on microvasculature could also be expected to be seen at the retinal vessels level as it has been shown that there is an active cholesterol metabolism at the retinal level, where low-density lipoprotein uptake is the main source of lipids in the retina [29,30]. Nevertheless, this effect has never been previously determined. Therefore, this study aims to investigate the correlation between oxysterols and retinal microvascular functional reactivity in apparently healthy individuals of various ages.

## 2. Materials and Methods

### 2.1. Study Participants

This study included healthy volunteers over 19 years old recruited through advertising at Aston University Vascular Research Laboratory (Birmingham, UK). The research was designed in accordance with the Declaration of Helsinki (2008) and ethical approval was sought from Aston University’s Ethics Committee. Written informed consent was received from all participants prior to their enrolment.

Study exclusion criteria were defined as the positive diagnosis of cardio/cerebrovascular diseases, peripheral arterial diseases, severe hypercholesterolemia/hypertriglyceridemia (defined as plasma triglycerides > 6.00 mmol/L or total cholesterol levels > 7.00 mmol/L), diabetes mellitus, as well as metabolic diseases or chronic etiologies that required treatment [3,7,20].

Potential participants on antihypertensive medications as well as individuals using other vasoactive drugs including dietary supplements were excluded from the study. Ocular diseases were also assessed, and participants were excluded from the study if they had signs of hypertensive retinopathy, a refractive error of more than ±3DS and more than ±1DC equivalent, intra-ocular pressure (IOP) greater than 21 mmHg, cataract, or any other media opacities, as well as a history of intra-ocular surgery or any form of retinal or neuro-ophthalmic disease affecting the ocular vascular system.

All the above investigations are standard for our lab [3,7,20].

### 2.2. General Investigations

All participants completed a general questionnaire that included details about their personal and family medical history, daily dietary habits, physical activities and alcohol consumption. 

Study measurements and examinations were conducted in the early morning following a 12 h overnight fast. Standard anthropometric measures of height and weight were recorded to determine body mass index (BMI = weight/height^2^).

### 2.3. Dynamic Retinal Vessel Analysis

All measurements were performed in a temperature-controlled environment (22 °C) following pupil dilation with 1% Tropicamide (Chauvin Pharmaceuticals Ltd., Kingstion upon Thames, UK).

**Figure 1:**
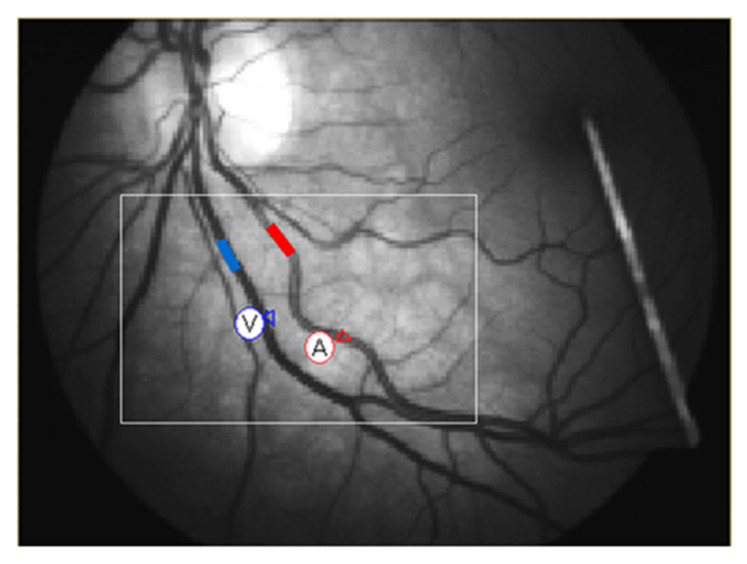
Example of retinal vessel selection before analysis.

**Figure 2:**
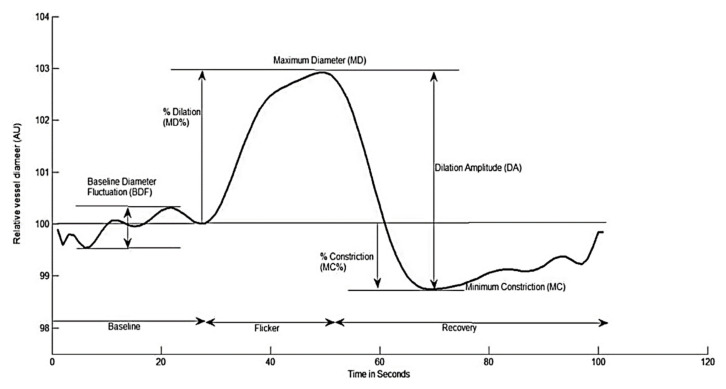
Graphical presentation of the dynamic retinal vessel response profile displaying the parameters calculated and used in analysis: AU, arbitrary units; BDF, baseline diameter fluctuation calculated as the maximum range in vessel diameter during first 30 s of baseline readings; MD, maximum dilation; MD%, percent dilation calculated as the percentage change in vessel diameter from baseline to maximum following onset of flicker; DA, dilation amplitude (difference between MD and MC during flicker); MC, maximum constriction; MC%, percent constriction calculated as the percentage change in vessel diameter from baseline to maximum following end of flicker.

Retinal vessel reactivity was assessed using the dynamic retinal vessel analyzer (DVA, IMEDOS GmbH, Jena, Germany) in accordance with an already established protocol (Figure 1) [4,31]. Only one randomly selected eye was measured. Figure 2 depicts the measured retinal vessel reactivity and time-course parameters averaged over the 3 cycles: [6,32].

### 2.4. Biomarkers Assays

Blood samples were collected in standard EDTA Vacutainer^®^ tubes. Fasting lipid panel included the four basic parameters: total cholesterol (CHOL), high-density lipoprotein cholesterol (HDL-C), low-density lipoprotein cholesterol (LDL-C) and triglycerides (TG) was assessed in all the participants using the Reflotron Desktop Analyser (Roche Diagnostics, UK). LDL-C values were calculated as per the Friedewald equation [33].

### 2.5. Measurement of Glutathione (GSH) and Oxidized Glutathione (GSSG)

Blood GSH and GSSG levels were analysed by the GSH recycling assay as detailed previously [7]. Briefly, 33.3 μL of 1 g/mL 5-sulfosalicylic acid (SSA) and 936.7 μL of stock buffer (125 mM sodium phosphate, 6.3 mM disodium EDTA pH 7.5) were mixed with 30 μL aliquot of EDTA blood. The mixture was vortexed followed by centrifugation at 13,000× *g* for 5 min. The supernatant was recovered and stored at −80 °C until further analysis within 2 months of collection [34]. GSH and GSSG levels were assessed by the GSR-DTNB recycling assay as we previously described [35]. The GSH levels [*t*-GSH−(2 × GSSG)] and the redox index (defined as the GSH/GSSG ratio) were determined. Since low-molecular weight thiols such as GSH are labile and sensitive to handling, special attention was given to blood collection, initial processing and storage as we described before [35]. All blood samples were collected in the morning (9–10 a.m.) and an aliquot of blood was processed (<15 min) for GSH assay immediately after collection.

### 2.6. Measurement of Nitric Oxide

Fasting venous blood samples were collected in citrated tubes. Samples were centrifuged at 3000 rpm for 15 min. The citrated plasma was then thawed and diluted 1:2 in 1X reagent diluent and levels of NO were measured in triplicates using Invitrogen nitric oxide assay kit^®^ (Bender Medsystems GmbH, Vienna, Austria).

### 2.7. Measurement of Interleukin-6 (IL-6)

Following the collection of fasted venous blood samples into citrate tubes, the samples were centrifuged at 3000 rpm for 15 min and the citrated plasma was then thawed and analysed for IL-6 levels using the commercially available Quantikine^®^ Human IL-6 enzyme-linked immunosorbent assay (ELISA) (R&D Systems, Inc., Minneapolis, MN, USA).

### 2.8. Extraction and Liquid Chromatography-Tandem Mass Spectrometry (LC-MS/MS) of Plasma Oxysterols

Oxysterols were extracted from 70 µL of human plasma spiked with 1 ng of deuterated internal standards (24-OHCd7, 25-OHCd6, 27-OHCd6, 7β-OHCd7, 7-KCd7). Briefly, plasmas were mixed with 430 µL methanol, vortexed and incubated on ice for 10 min in the presence of 4 mg/mL BHT before centrifugation at 14,000× *g* for 10 min. The methanolic supernatant was diluted with acidified water of up to 10% methanol for loading onto an SPE cartridge. Oxysterols were eluted with 1.8 mL of butyl acetate and dried under a vacuum as described by Dias et al. [36]. Percentages of process recovery ± standard deviation for internal oxysterol standards were between 72.04 ± 11.6 and 81.80 ± 8.6.

The lipid extracts were dissolved in 40 μL of 40:60 Methanol: H_2_O and analyzed with a liquid chromatography UltiMate 3000 HPLC system (Dionex, Thermo Scientific Ltd., UK) coupled on-line with an electrospray tandem triple quadrupole-linear ion trap mass spectrometer (QTrap 5500, ABSciex) as described previously (Dias et al., 2018), with some modifications. Briefly, the lipid extracts were separated using a reverse phase C18 column (100 × 3.2 mm, 5.0 μm particle size; Macherey-Nagel) with mobile phases A (70:30 Methanol: H_2_O with 0.1% formic acid) and B (90:10 Isopropanol:Methanol with 0.1% formic acid). The flow rate was 200 μL/min and the column was maintained at 45 °C.

### 2.9. Sample Size and Statistical Analysis

Based on previous studies, a change of 30% with an SD of 2.5% in retinal vessels reactivity was shown to be significant [37]. As the study design was multifactorial in nature, it was calculated that a sample size of *n* = 42 was sufficient to provide 95% power at an alpha level of 0.05.

All analyses were performed using Statistica^®^ software (version 13.3, StatSoft Inc., Tusla, OK, USA). Distributions of continuous variables were determined by the Shapiro-Wilks test. In cases where the normality of the data could not be confirmed, appropriate data transformations were made, or non-parametric statistical alternatives were used. Univariate associations were determined using Pearson’s (normally distributed data) or Spearman’s method (non-normally distributed data) and forward stepwise multiple regression analyses were performed to test the influence of clinical parameters and circulating markers on the measured vascular reactivity variables. Differences between groups were subsequently assessed using one-way ANOVA or ANCOVA, as appropriate, followed by Tukey’s post hoc analysis. *p*-values of <0.05 were considered significant [3,7,20].

## 3. Results

### 3.1. Clinical Characteristics

A total number of 56 participants were initially screened for study inclusion, 14 of which were excluded based on the quality of retinal vascular image analysis. The remaining 42 participants were included in the final analysis and classified into three age groups. Group 1: 19–30 years (10 male, 6 female); Group 2: 31–50 years (eight male, eight female); and Group 3: 51–70 years (eight male, eight female). The general characteristics of the study population are presented in Table 1. There were no significant differences in sex, BMI, heart rate (HR), IOP, glucose, TG and HDL-C between the study groups (all *p* > 0.05). Although still within the normal range, older participants had higher CHOL and LDL-C values than younger ones, albeit close to the upper limit for these parameters (*p* = 0.0071 and 0.0112, respectively) (Table 1). Similarly, LDL-C/HDL-C ratio was significantly higher in the older group compared to the young and middle age group (*p* = 0.0370 and 0.0082, respectively).

### 3.2. Retinal Microvascular Function

After controlling all the influential covariates identified using multivariate analysis, there were no significant differences between the study groups with regard to arterial baseline, baseline corrected flicker response (BCFR), maximum dilation (MD), maximum constriction (MC), time to maximum constriction (tMC), arterial dilation slope (Slope_AD_) and all venous microvascular parameters (all *p* > 0.05, Table 2 and Table 3).

**Figure 3:**
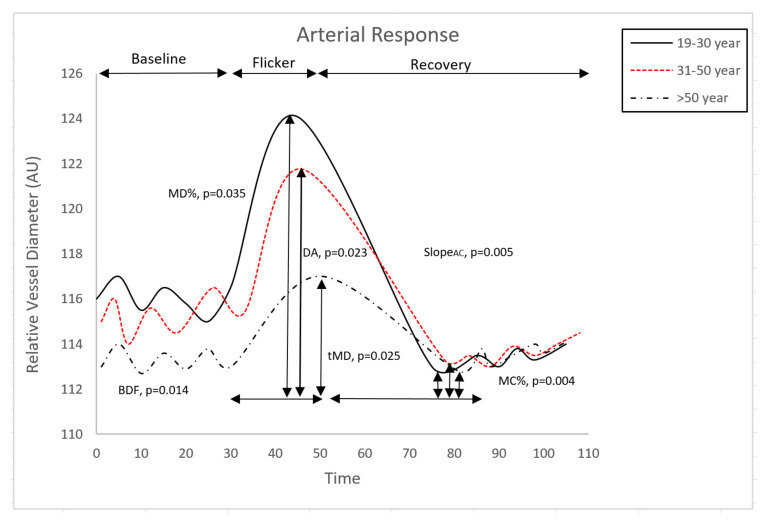
Comparison of retinal arterial response profile across groups. Abbreviations: AU, arbitrary units; BDF, baseline diameter fluctuation calculated as the maximum range in vessel diameter during first 30 s of baseline readings; MD%, percent dilation calculated as the percentage change in vessel diameter from baseline to maximum following onset of flicker; tMD, time to reach maximum diameter during flicker; slope_AC_, slope of arterial constriction calculated as (MC-MD)/(tMC); DA, dilation amplitude (difference between MD and MC during flicker); MC%, percent constriction calculated as the percentage change in vessel diameter from baseline to maximum following end of flicker.

However, the post hoc analysis revealed a significantly decreased baseline diameter fluctuation (BDF), maximum dilation percentage (MD%), maximum constriction percentage (MC%), dilation amplitude (DA) and arterial dilation slope (Slope_AC_) with age (*p =* 0.0140, *p =* 0.0360, *p =* 0.0040, *p =* 0.0230 and *p =* 0.0055, respectively, Table 2, Figure 3). Moreover, artery time to maximum dilation (tMD) increased significantly with age (*p =* 0.0254, Table 2).

No statistically significant differences were found between retinal venous vascular function parameters among the study groups (Table 3).

### 3.3. Oxidative Stress and Inflammatory Markers

After controlling all the influential covariates identified using multivariate analysis, post hoc analysis showed a significant increase in 7-KC, 25-OHC, 27-OHC, 7β-OHC and 7,25-OHC with age (*p* = 0.0051, *p* = 0.0067, *p* = 0.0192, *p* = 0.0168, *p =* 0.0113, respectively). Post hoc analysis did not reveal any significant differences in IL-6 levels among the three age groups (Table 4). However, the GSH/GSSG ratio decreased significantly with age among the study population (*p* = 0.0279). No other significant differences were recorded (all *p* > 0.05, Table 4).

No statistically significant differences were found in the NO concentrations between the study groups (all *p* > 0.05).

### 3.4. Correlations between Vascular and Systemic Circulatory Parameters

Univariate analysis revealed that blood 7-KC, 25-OHC and 7β-OHC levels correlated significantly and positively with tMD in all the study participants (r = 0.4318, *p* = 0.025; r = 0.2153, *p* = 0.042; r = 0.4029, *p* = 0.037, respectively). 

Additionally, 25-OHC and 7β-OHC were negatively correlated to arterial MD% (r = −0.3879, *p* = 0.046; r = −0.5324, *p* = 0.004, respectively). Similarly, 27-OHC and 7β-OHC significantly correlated negatively with artery Slope_AC_ (r = −0.2291, *p* = 0.0250; r = −0.4576, *p* = 0.016, respectively).

There were no correlations between the GSH redox ratio and retinal vascular parameters in any of the study groups (all *p* > 0.05). However, GSH/GSSG levels correlated significantly and negatively with 7β-OHC and 25-OHC (r = −0.5129, *p* = 0.021; r = −0.4799, *p* = 0.032, respectively).

## 4. Discussions

In the present study, we have examined the relationship between circulatory markers of oxidative stress and endothelial function with dynamic retinal microvascular changes in various age groups. Our results show that, with age, oxysterol levels change in parallel with microvascular function and with age in individuals without clinically overt CVDs.

In accordance with the existing literature, our results demonstrated an ageing-dependent systematic increase in plasma circulatory lipids [38] and oxidative stress markers [39,40]. In addition, similar to previous research, circulating oxysterols concentrations were also increased with age [41,42,43]. Another consistency with previously published work was the fact that our healthy and yet older individuals (above 50 years old) displayed abnormal microvascular function in comparison to younger and middle-aged participants [44,45]. Indeed, it is well known that healthy and young endothelium usually retains antithrombotic, vasodilatory, anti-inflammatory and antioxidant properties, which regulate the vessels response to vasodilatory stimulants [46]. One hallmark of endothelium dysfunction is the progressively impaired vasodilatory response to blood flow and vasodilating compounds [47]. It was believed that reduced vasodilation, even at the retinal level, is mainly due to a diminished bioavailability of NO. Nevertheless, in our study, the NO levels were not different between the three groups and the impaired microvascular responses were only correlated to abnormal plasma oxysterols levels. Although a surprise, it is possible that these circulatory biomarkers are, indeed, the initial elusive culprits for the consistently observed age-related retinal vascular dysfunction and not early abnormal NO levels [6]. This observation and hypothesis do not eliminate, however, the well-demonstrated involvement of NO in early microvascular dysfunction. Indeed, oxysterols are involved in the impairment of endothelial-eNOS-dependent NO generation. Moreover, as well-known ROS inducers, oxysterols also contribute to a decline in NO availability [48]. In healthy ageing, NO production or bioavailability may not be impaired, and it may be too early to detect any changes to cytokine release by endothelial cells. Therefore, the fact that NO levels or IL-6 levels were not different between the three groups of individuals suggests that the oxysterol actions were the first to appear and be measured. However, this study did not look into the underlying biological mechanisms by which oxysterols change vascular reactivity and further research will be necessary to confirm our hypothesis.

In line with previous reports, our study shows that the LDL-C/HDL-C ratio is higher in the older population. As the oxidative stress increases with age, LDL lipids and proteins can undergo oxidative modifications. Oxidized LDL is regarded as a representative parameter of oxidative stress and endothelial dysfunction [49]. We have previously shown that oxidized LDL-lipids are more damaging to endothelial barrier properties and increase the release of inflammatory cytokines by endothelial cells [50]. As noted, circulating oxysterols concentrations were strongly and positively correlated to plasma total cholesterol and LDL-C concentrations. Levels of 7-KC, 25-OHC, 27-OHC, 7β-OHC and 7,25-OHC increased with age, particularly after the age of 30, with the highest increase observed in the above 50-year group. Similarly, both total cholesterol and LDL-C levels were higher in middle-aged and elderly individuals compared to younger participants.

Oxidative stress is known to be a major driving force in ageing and in the development of CVDs. In addition, oxysterols may contribute to endothelial senescence and CVDs by making the endothelial cells more susceptible to various risk factors for CVDs [28]. Therefore, our observations are highly significant and emphasis the fact that the assessment of plasma oxysterols, as well as the retinal microvascular dysfunction, is among the earliest markers for CVDs risk.

## 5. Conclusions

To date, the clinical tools used to assess the future risk for CVDs rely mainly on structural vascular changes and, therefore, as a direct result, either overestimate or underestimate individualized risk [51]. Our observations suggest that using DVA and plasma oxysterols levels, instead of other time consuming and less sensitive methods, could increase the sensitivity of CVDs risk prediction, especially in individuals free of clinical overt CVDs but with various risk factors. Additionally, as molecular and imaging biomarkers drive the shift towards personalized medicine, DVA and oxysterol levels can be used as a quick, minimally invasive method of profiling individualized vascular risk to be used in prediction, prevention, and personalized intervention.

## Figures and Tables

**Table 1:** Summary of the systemic characteristics of the study participants.

Variable	(19–30) Year	(31–50) Year	(51–70) Year	*p*-Value	Post-Hoc
Number	16 10M:6F	16 8M:8F	10 4M:6F	-	-
Age (years)	24.54 (1.74)	37.81 (1.57)	60.75 (2.22)	0.0001 *	1 < 2 < 3
SBP (mmHg)	115.27 (3.80)	114.7 (3.95)	118.5 (5.10)	0.82946	-
DBP (mmHg)	64.64 (2.54)	70.9 (2.67)	72.5 (3.44)	0.13002	-
HR (bpm)	63.17 (3.29)	68.1 (2.55)	66.18 (2.43)	0.50522	-
IOP (mmHg)	12.93 (0.57)	13.23 (0.60)	14.83 (0.80)	0.14723	-
BMI (kg/m^2^)	23.34 (1.13)	26.02 (1.19)	26.59 (1.53)	0.16039	-
Glucose (mmol/L)	4.41 (0.21)	4.99 (0.28)	4.68 (0.28)	0.26740	-
TG (mmol/L)	0.83 (0.065)	1.0 (0.081)	0.96 (0.088)	0.22376	-
CHOL (mmol/L)	3.95 (0.17)	4.14 (0.21)	4.93 (0.24)	0.0071 *	1 < 2 < 3
HDL-C (mmol/L)	1.33 (0.08)	1.44 (0.10)	1.25 (0.12)	0.50590	-
LDL-C (mmol/L)	2.28 (0.17)	2.60 (0.21)	3.01 (0.24)	0.01116 *	1 < 2 < 3
LDL-C/HDL-C	1.64 (0.13)	1.85 (0.2)	2.37 (0.19)	0.0658	1 = 2 3 > 1,2

Abbreviations: SBP, systolic blood pressure; DBP, diastolic blood pressure; HR, heart rate; IOP, intraocular pressure; BMI, body mass index; TG, triglycerides; CHOL, total cholesterol; HDL-C, high-density lipoprotein cholesterol; LDL-C, low-density lipoprotein. * Significant *p*-values are indicated where *p* < 0.05 was considered significant.

**Table 2:** Summary of retinal arterial vascular function parameters.

Variable	(19–30) Year	(31–50) Year	(51–70) Year	*p*-Value Anova/Ancova	Post-Hoc
Baseline	117.44 (4.24)	116.60 (4.45)	113.41(5.74)	0.61902	
BDF	7.06 (0.65)	5.92 (0.70)	3.49 (0.88)	0.0140 *	1 > 2 > 3
BCFR	4.90 (0.72)	4.81 (0.80)	2.54 (0.90)	0.1058	-
MD	124.94 (4.13)	121.76 (4.60)	117.02 (5.20)	0.4986	-
tMD	14.67 (0.77)	16.15 (0.73)	19.29 (0.83)	0.0254 *	1 < 2 < 3
MD %	5.64 (0.63)	4.50 (0.70)	3.24 (0.79)	0.0360 *	1 > 2 > 3
MC	112.99 (4.32)	113.24 (4.78)	112.80 (5.42)	0.5985	-
tMC	25.39 (2.34)	28.93 (2.60)	27.523 (2.93)	0.5974	-
MC%	−3.85 (0.36)	−2.43 (0.4)	−1.70 (0.46)	0.0040 *	1 > 2 > 3
DA	11.95 (1.27)	9.74 (1.40)	6.04 (1.60)	0.0230 *	1 > 2 > 3
Slope_AD_	0.52 (0.11)	0.51 (0.12)	0.26 (0.14)	0.3030	-
Slope_AC_	−0.62 (0.062)	−0.38 (0.07)	−0.30 (0.08)	0.0055 *	1 > 2, 2 = 3, 1 > 3

Abbreviations: ANOVA, analysis of variance; ANCOVA, analysis of covariance; Baseline, baseline diameter; BDF, baseline diameter fluctuation; BCFR, Baseline corrected flicker response; MD, maximum dilation; tMD, time to reach MD; MD%, percent dilation; MC, maximum constriction; tMC, time to reach MC; MC%, percent constriction; DA, dilation amplitude (difference between MD and MC during flicker); Slope_AD_, slope of arterial dilation; Slope_AC_, slope of arterial constriction. * Significant *p*-values are indicated where *p* < 0.05 was considered significant.

**Table 3:** Summary of retinal venous vascular function parameters.

Variable	(19–30) Year	(31–50) Year	(51–70) Year	*p*-Value Anova/Ancova	Post-Hoc
Baseline	142.47 (4.25)	143.55 (4.70)	131.39 (5.33)	0.19389	-
BDF	5.45 (0.75)	5.02 (0.84)	5.61 (0.95)	0.88379	-
BCFR	3.00 (0.85)	4.87 (0.95)	3.14 (1.07)	0.30859	-
MD	149.09 (4.44)	150.26 (4.91)	138.19 (5.60)	0.22687	-
tMD	23.76 (1.47)	19.37 (1.63)	21.95 (1.85)	0.15744	-
MD %	4.66 (0.66)	4.75 (0.74)	5.15 (0.83)	0.89666	-
MC	140.64 (4.24)	140.37 (4.69)	129.44 (5.32)	0.22113	-
tMC	32.12 (2.16)	34.41 (2.40)	33.71 (2.71)	0.76678	-
MC%	−1.30 (0.45)	−2.20 (0.49)	−1.50 (0.56)	0.38622	-
DA	8.45 (1.36)	9.90 (1.50)	8.75 (1.70)	0.76728	-
Slope_VD_	0.33 (0.046)	0.36 (0.051)	0.35 (0.06)	0.86531	-
Slope_VC_	−0.60 (0.12)	−0.31 (0.13)	−0.39 (0.14)	0.22394	-

Abbreviations: ANOVA, analysis of variance; ANCOVA, analysis of covariance; Baseline, baseline diameter; BDF, baseline diameter fluctuation; BCFR, Baseline corrected flicker response; MD, maximum dilation; tMD, time to reach MD; MD%, percent dilation; MC, maximum constriction; tMC, time to reach MC; MC%, percent constriction; DA, dilation amplitude (difference between MD and MC during flicker) Slope_VD_, slope of venous dilation; Slope_VC_, slope of venous constriction.

**Table 4:** Summary of normalized oxidative stress and inflammatory systemic circulatory markers.

Variable	(19–30) Year	(31–50) Year	(51–70) Year	*p*-Value Anova/Ancova	Post-Hoc
7-KC (nmol/mmol)	10.49 (1.71)	13.28 (1.17)	17.26 (1.17)	0.0051 *	1 < 2 < 3
25-OHC (nmol/mmol)	16.42 (3.86)	20.86 (2.65)	30.56 (2.65)	0.0067 *	1 < 2 < 3
27-OHC (nmol/mmol)	16.61 (3.05)	20.55 (2.09)	26.79 (2.09)	0.0192 *	1 < 2 < 3
4β-OHC (nmol/mmol)	1.02 (0.35)	0.81 (0.24)	1.14 (0.24)	0.6054	-
7β-OHC (nmol/mmol)	6.65 (1.70)	9.86 (1.17)	12.74 (1.17)	0.0168 *	1 < 2 < 3
7,27-OHC (nmol/mmol)	8.45 (2.30)	8.64 (1.57)	10.65 (1.57)	0.5986	-
7,25-OHC (nmol/mmol)	5.60 (2.60)	8.89 (1.78)	11.41 (1.80)	0.0113 *	1 < 2 < 3
NO (uM)	46.45 (4.37)	43.02 (3.39)	39.73 (3.10)	0.4495	
IL-6 (pg/mL)	3.23 (0.45)	3.24 (0.31)	3.09 (0.31)	0.9424	-
GSH/GSSG	27.25(7.95)	20.84 (7.95)	17.05 (8.60)	0.0279 *	1 > 2 > 3

Abbreviations: ANOVA, analysis of variance; ANCOVA, analysis of covariance; 7-KC, 7-Ketocholesterol; 25-OHC, 25-Hydroxycholesterol; 27-OHC, 27-Hydroxycholesterol; 4β-OHC, 4β-Hydroxycholesterol; 7β-OHC, 7β-Hydroxycholesterol; 7,27-OHC, 7,27-Hydroxycholesterol; 7,25-OHC, 7,25-Hydroxycholesterol; NO, nitric oxide; IL-6, interleukine-6; GSH, glutathione; GSSG, Glutathione disuphide; GSH/GSSG, redox ratio. * Significant *p*-values are indicated where *p* < 0.05 was considered significant.

## Data Availability

All of the data is contained within the article.
